# Hernia Reduced by Distension

**Published:** 1874-11

**Authors:** M. Perl


*MEDICAL AND SURGICAL REPORTER.—October,* 1874:

STRANGULATED HERNIA REDUCED BY FORCING AIR INTO THE INTESTINES—*By Dr. M. Perl.*—I was called, on September 20th, at 11 P.M., to see A. R., æt. 51, machinist, who had been suffering for four days with strangulated inguinal hernia, left side. He had been on a big spree, as his room-mate told me, and had lost his truss. Pulse very feeble, 120; tongue, thick brown coating, very dry; vomits everything he takes.

After trying for six hours to reduce the hernia, without success, using enemas with tobacco infusion, and everything I could think of, I went after my instruments, and made all necessary preparations for the operation, only waiting the return of a messenger with some chloroform, when it occurred to me to force air into the intestines through the anus.

In my bag I had a Richardson's apparatus for local anæsthesia, to the bellows of which I attached a small tube, inserted it in the anus, and commenced to force in air. The lower part of the bowels soon distended, and after twenty minutes of constant working of the bellows, the patient became uneasy. I withdrew the tube, which was followed by some flatus. I then laid the patient on an inclined plain, lower extremities higher than the trunk, which I had used before also, and tried the taxis again, when, to my great satisfaction, and the patient's delight, a quantity of gases escaped, and the hernia was instantly reduced.

I then ordered an enema with soap and oil, which produced a copious discharge; the first in five days. The pulse soon went down to 90; he drank a cup of coffee, which he retained; is at work now, and will take care not to lose his truss again.

